# Physiological Sensor Modality Sensitivity Test for Pain Intensity Classification in Quantitative Sensory Testing

**DOI:** 10.3390/s25072086

**Published:** 2025-03-26

**Authors:** Wenchao Zhu, Yingzi Lin

**Affiliations:** Intelligent Human Machine Systems Laboratory, Department of Mechanical and Industrial Engineering, Northeastern University, Boston, MA 02155, USA

**Keywords:** machine learning, pain intensity, physiological signals, quantitative sensory testing, sensor modality, time window

## Abstract

Chronic pain is prevalent and disproportionately impacts adults with a lower quality of life. Although subjective self-reporting is the “gold standard” for pain assessment, tools are needed to objectively monitor and account for inter-individual differences. This study introduced a novel framework to objectively classify pain intensity levels using physiological signals during Quantitative Sensory Testing sessions. Twenty-four participants participated in the study wearing physiological sensors (blood volume pulse (BVP), galvanic skin response (GSR), electromyography (EMG), respiration rate (RR), skin temperature (ST), and pupillometry). This study employed two analysis plans. Plan 1 utilized a grid search methodology with a 10-fold cross-validation framework to optimize time windows (1–5 s) and machine learning hyperparameters for pain classification tasks. The optimal time windows were identified as 3 s for the pressure session, 2 s for the pinprick session, and 1 s for the cuff session. Analysis Plan 2 implemented a leave-one-out design to evaluate the individual contribution of each sensor modality. By systematically excluding one sensor’s features at a time, the performance of these sensor sets was compared to the full model using Wilcoxon signed-rank tests. BVP emerged as a critical sensor, significantly influencing performance in both pinprick and cuff sessions. Conversely, GSR, RR, and pupillometry demonstrated stimulus-specific sensitivity, significantly contributing to the cuff session but with limited influence in other sessions. EMG and ST showed minimal impact across all sessions, suggesting they are non-critical and suitable for reducing sensor redundancy. These findings advance the design of sensor configurations for personalized pain management. Future research will focus on refining sensor integration and addressing stimulus-specific physiological responses.

## 1. Introduction

Pain is a complex and subjective experience, remaining one of the most significant clinical challenges, with 51.6 million U.S. adults (20.9%) experiencing chronic pain and 17.1 million (6.9%) suffering from high-impact chronic pain during 2021 [1,2]. The symptom of chronic pain causes the greatest source of disability for human beings, leading to substantial issues and affecting the quality of life for individuals and society [3,4].

Pain can be understood as a conscious interpretation of sensory stimuli that triggers nociceptive afferents, accompanied by the mental projection of these stimuli onto specific body regions. Pain assessment involves approximating an individual’s subjective self-report, which serves as their ground truth [5,6]. The traditional pain assessment is performed through a survey based on participants’ subjective perception of their pain, such as the numeric rating scale (NRS), the visual analogue scale (VAS), and the verbal rating scale (VRS). However, self-reported assessments are prone to bias from anxiety, memories, pain intensity, and physical activities [6,7,8]. The consequences of such inaccuracies can lead to under-treatment and over-treatment of pain that is either ineffective or detrimental to patient safety [9,10].

Quantitative Sensory Testing (QST) was developed as an objective, standardized way to evaluate pain sensitivity and pain perception using calibrated mechanical or thermal stimuli to measure sensory thresholds and tolerances [11]. This technique can aid in diagnosing conditions such as neuropathic pain and chronic low back pain by detecting abnormalities in QST sessions. For example, one QST session, the cuff inflation test, has shown that cLBP patients require lower cuff pressure to evoke moderate pain compared with healthy controls, and they also rate mechanical probes as more painful [12]. In addition, psychosocial factors—including emotional states and pain catastrophizing—further influence nociceptive processing and contribute to variations in pain perception [13,14].

Physiological sensors have developed as an objective measurement of human states and characteristics [15,16,17], such as blood volume pulse (BVP), electroencephalogram (EEG), galvanic skin response (GSR), respiration rate (RR), electromyography (EMG), skin temperature (ST), and pupillometry. For example, increased skin conductance level in GSR was detected when external noxious stimuli (e.g., pressure, thermal, cold pain) were presented [18]. Heart rate and heart rate variability, which can be derived from BVP signals, is associated with a stress response. During different pain stimuli, a decreased BVP or an increase in heart rate have been observed [19,20]. ST can be measured in the palm and the back of the hand, and decreased ST has been reported during and after painful stimuli [20,21]. EEG studies have addressed the correlations between noxious stimuli and different EEG frequency bands. For example, decreases in the alpha band have been observed as the common indicator [22,23].

Research has demonstrated the potential of using sensors in classifying pain intensity levels [24,25,26]. For example, Guo et al. estimated three levels of cold pain using facial expression by comparing three neural network models, and the personalized spatial–temporal framework using a convolutional long short-term memory model achieved the highest performance [27]. Another study measured the pain level via features generated from the pupillometry data using a genetic algorithm with an artificial neural network classifier, and the best performance was obtained with an accuracy of 81% [28]. EEG studies have demonstrated statistical differences in central and occipital regions, and was able to classify pain and no-pain states using multi-layer CNN frameworks [29,30]. Multimodal physiological classification with decision-level fusion and feature-level fusion proved promising in pain level detection and classification [21,31,32].

Combining multimodal physiological sensors with QST represents a frontier in pain research, enhancing the objectivity and sensitivity of pain assessments. BVP signals have been used to classify the pressure session, achieving 96.6% accuracy in a binary (threshold vs. tolerance) task [33]. GSR has assessed conditioned pain modulation between patient and healthy groups, revealing significant differences in the dominant hand (*p* = 0.003) [34]. EMG signals were evaluated under varying cuff pressures [35]. Despite these advances, few studies have examined comprehensive pain-level classification across all QST sessions. Additionally, the sensitivity of each sensor to these classifications remains largely unexplored.

Time window selection influences the interpretation of physiological signals in the task of pain assessment. One study demonstrated two distinct labeling approaches: fixed time windows and percentage-based timestamps. The fixed time window method segments data in a consistent and fixed manner to provide a straightforward approach for analyzing responses [36,37]. In contrast, percentage-based timestamping aligns labels to the individual’s pain threshold and tolerance tailored to personal variations in pain perception [25]. Importantly, the chosen segmentation method directly impacts the number of samples generated for analysis and influences the dataset size available for machine learning models.

While multimodal physiological signals can aid in QST pain assessment tasks, two critical gaps remain in the current literature. First, existing studies have primarily focused on isolated noxious stimuli (e.g., cold pain, pressure, or cuff pressure) but rarely compared them across different noxious stimuli in a holistic way. Secondly, existing studies either combine all sensor data into the analysis model or exclusively analyze one of the modalities. The relative contributions of individual sensors remain unclear. Furthermore, the time window of signal segmentation under different tasks needs to be cautiously selected. Our study aims to advance the understanding of physiological sensor contributions to pain assessment and the development of individualized pain biomarkers, by addressing two research questions:

- The first question is to quantify the sensitivity of different time windows and machine learning classification model selection for pain level classification.
- The second question is to evaluate how excluding individual physiological sensors affects the model performance.

## 2. Materials and Methods

### 2.1. Participants

The study was conducted from January to May 2022 and approved by the Brigham and Women’s Hospital Institutional Review Board (IRB), Boston, MA, USA (protocol code 2019P002781, 18 November 2019). Healthy participants and chronic low back pain patients who have had cLBP for at least three months with an average intensity of more than three out of ten on pain scales were recruited. All participants were neurologically intact and had no history of myocardial infarction, substantial motor or sensory deficits, or no evidence of cognitive impairment.

### 2.2. Apparatus

The study used sensors to monitor physiological responses during QST sessions. Pupillometry data were tracked using Tobii Pro Glasses 2 (Tobii, Danderyd, Sweden). Other sensors (FlexComp Infiniti, Thought Technology, Montreal, QC, Canada) that measured the participant’s physiological responses included a BVP (SA9308M, Thought Technology) sensor for heart rate tracking through the middle finger of the non-dominant hand, a chest-mounted respiration sensor (SA9311M, Thought Technology), an EMG sensor (T9306M, Thought Technology) for muscle activity on the non-dominant forearm, an ST sensor (SA9310M, Thought Technology) on the back of the non-dominant hand, and a GSR sensor (SA9309M, Thought Technology) for electrical activity between the index and ring fingers on the non-dominant hand. A computer system was used to collect and store data (Dell Latitude E6230, Dell, Round Rock, TX, USA).

### 2.3. Experimental Procedures

**Figure 1:**
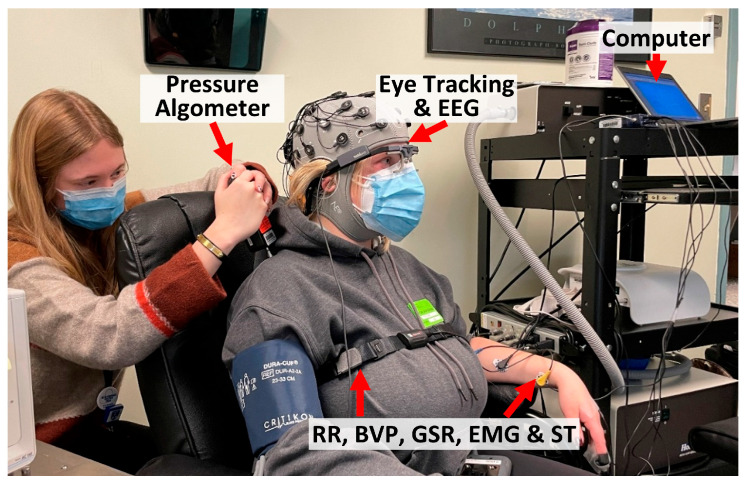
Experiment apparatus. In this pressure pain experiment, a digital pressure algometer was applied on the participant’s trapezius. Physiological signals (RR, BVP, GSR, EMG, ST, and ET) were collected in the meantime.

Participants were familiarized with the QST equipment and completed the Brief Pain Inventory questionnaire [38]. The physiological data collection for each participant took approximately 80–120 min. As shown in Figure 1, the process involved the following:

(1) Participants were seated comfortably in a reclining chair.
(2) A research assistant helped participants wear all sensors, including pupillometry, BVP, GSR, EMG, ST, and RR. The setup took around 20 min.
(3) A one-minute baseline was recorded, during which the participant stayed in a natural resting condition.
(4) Data collection occurred over 30 min for one round of QST, during which participants followed instructions from the research assistant, reported pain intensities, and were asked to minimize unnecessary movement.
(5) Another one-minute baseline was recorded.
(6) Participants then performed physical maneuvers spanning about 3–5 min, with sensors disconnected.
(7) Participants then repeated steps 3 to 5 for a second round of QST collection.
(8) The sensors were removed, and participants were debriefed and compensated.

Due to COVID-19 safety measures, all research staff and study participants were required to wear a face covering/mask to cover the nose and mouth, and only four people were present in the testing room at one time due to the COVID-19 period.

### 2.4. Quantitative Sensory Testing

QST has four sessions: pressure pain threshold and tolerance, temporal summation of mechanical pinprick pain, temporal summation of cuff pain, and conditioned pain modulation. The Temporal Summation of Pain tests the ability of the central nervous system to amplify the incoming pain over time when applying an increasing pain. It can be demonstrated in various pain modalities, including mechanical pinprick and cuff pain.

(1) Pressure pain threshold and tolerance were assessed using a digital pressure algometer. The testing sites were located on the dorsal surface of the forearm and over the trapezius muscle in the upper back and neck region. The researcher increased the pressure pain gradually via a flat round transducer on a small skin area (probe area 0.785 cm^2^) at a steady speed of ~1 lb./s (0.45 kg/s). The pressure value was first recorded when the participant reported the onset of pain as a pressure pain threshold and was terminated when the participant reached their maximum pain tolerance. Four trials were performed, including the left forearm, the right forearm, the left trapezius, and the right trapezius.
(2) Mechanical pinprick pain was assessed by applying 10 calibrated force pinprick stimuli to the skin at a fixed frequency (1 Hz). Participants were asked to rate their pain intensity after the 1st, 5th, and 10th stimuli. The procedure was first applied on the left index finger and then repeated on the right index finger.
(3) Cuff pain was assessed by inflating a blood pressure cuff on the left leg to a threshold pressure level (5 out of 10 on a scale) and maintained for a fixed duration (2 min). Participants were asked to rate their pain levels every 30 s.
(4) Conditioned pain modulation was assessed by applying a noxious thermal stimulus and an increasing pressure pain simultaneously. Participants were first asked to submerge their dominant hand into the cold-water bath set at 6 degrees Celsius. Meanwhile, increasing pressure was applied to the non-dominant trapezius muscle, as described in the pressure pain steps. The participants then reported their onset of pain and their maximum pain tolerance. The post-pain rating was registered 15 s after the cessation of pressure pain.

### 2.5. Data Preprocessing

**Figure 2:**
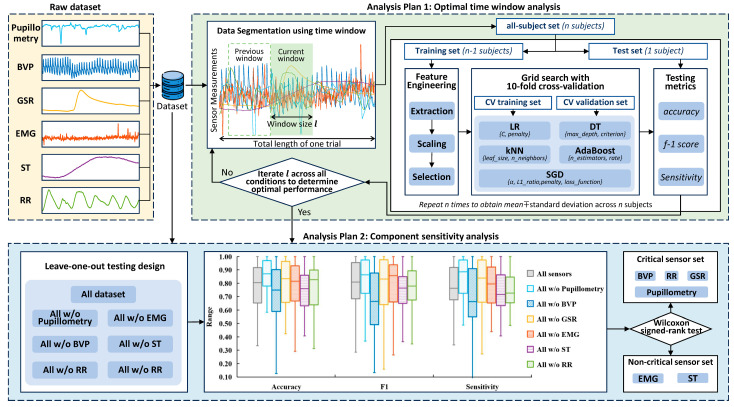
Diagram of the study. After collecting raw datasets from multimodal physiological sensors (BVP: blood volume pulse, GSR: galvanic skin response, EMG: electromyography, ST: skin temperature, RR: respiration rate), the dataset underwent two analysis plans: (1) perform the optimal time window analysis to select the optimal time window and hyperparameters via grid search. Time windows included 1, 2, 3, 4, and 5 s; (2) undergo component sensitivity analysis to investigate the performance across 7 distinct leave-one-out sets.

The overall research diagram is presented in Figure 2. First, physiological (BVP, GSR, EMG, ST, RR, and pupillometry) data were synchronized by resampling them to 50 Hz. The left-eye and right-eye pupillometry data were interpolated to fill in any missing gaps [20]. The BVP signal was filtered via a fifth-order Butterworth band-pass with [0.5, 12] Hz as cut-off frequencies. The GSR was filtered via a fifth-order 1 Hz low-pass Butterworth filter. The RR was filtered via a fifth-order Butterworth band-pass with [0.1, 1] Hz as cut-off frequencies. In addition, eight time-series HRV data were generated from BVP signals, including PPG rate, meanNN, SDNN, RMSSD, SDSD, HF, SD1, and SD2 using the NeuroKit2 package in Python 3.7.9 [28]. For extracting heart rate variability signals, a 15 s sliding time window with 50 Hz was selected.

### 2.6. Feature Extraction and Selection

Features were extracted from all physiological sensors. GSR signals were separated into phasic and tonic signals. Statistical features were then generated from all physiological sensors, such as mean, median, range, variance, standard deviation, skewness, and kurtosis [39]. Five additional features were generated from EMGs, including mean absolute value, root mean square, variance, zero crossings, waveform length, and slope signal changes [39].

Principal Component Analysis was utilized for feature selection by setting a 90% information variance threshold to determine the cumulative features to be used.

### 2.7. Analysis Plan

To solve the research questions, a two-phase analysis plan was employed.

Analysis Plan 1 (optimal time window analysis): this phase focused on determining the optimal time window for signal segmentation and evaluating the performance of various machine learning models across all QST sessions. The time window candidates included 1 s, 2 s, 3 s, 4 s, and 5 s. A grid search methodology was employed to explore the relationship between time window lengths and classification performance, using a 10-fold cross-validation framework. The output from Plan 1 was to identify the combination of time window and model hyperparameters that achieved the highest accuracy, F1 score, and sensitivity for each QST session.

Analysis Plan 2 (component sensitivity analysis): this phase investigated the individual contribution of each sensor modality to classification performance. Using a leave-one-out (LOO) iteration strategy, one sensor’s features were excluded at a time, and the model was retrained and tested using the remaining sensors. The performance of each LOO model was compared to the full model using statistical analysis (i.e., Wilcoxon signed-rank test).

The Wilcoxon signed-rank test is a nonparametric statistical test to compare two samples. This is a useful alternative to the paired *t*-test when the data do not follow a normal distribution. The differences between paired observations were computed and their absolute values were ranked. The test statistic was derived by summing the ranks of the positive and negative differences. Sensors with significant differences in Wilcoxon signed-rank test statistics (i.e., *p* value is below 0.05) were identified as critical sensor candidates. Sensors with minimal impact were identified as non-critical sensor candidates. The optimal time window and the optimal machine learning model were predetermined from Analysis Plan 1’s results.

The classification task was to predict pain intensity states from physiological features and compare them with subjective ratings as the ground truth. In the pressure pain test, a three-class classification task was used to differentiate among three pain states: baseline (no pain), threshold (threshold of pressure), and tolerance (tolerance of pressure). This classification was applied to data combining pressure and conditioned pain modulation sessions, as both sessions employed identical labels for pressure threshold and tolerance. The classification task in both the pinprick session and cuff session was classifying pain intensity states based on numerical rating scales (0–10). Participants’ self-reports were categorized into three levels: 1–3 (Mild Pain), 4–6 (Moderate Pain), and 7–10 (Severe Pain). This classification was conducted separately for stimuli applied to the left and right hands. Finally, the third task aimed to classify the pain intensity levels during a 2-minute temporal summation of cuff sensations. Similar to the pinprick classification task, the pain levels were categorized as Mild, Moderate, and Severe.

A grid search approach involving five classification models and their respective hyperparameters (detailed in Table 1) was used. These models included logistic regression (LOG), decision tree (DT), k Nearest Neighbors (KNN), Stochastic Gradient Descent (SGD), and AdaBoost (ADA). The Synthetic Minority Oversampling Technique (SMOTE) was applied to the training dataset to balance the minority classes [40]. Principal Component Analysis (PCA) was selected to reduce dimensionality with an 80% threshold.

## 3. Experimental Results

Detailed demographic information is presented in Table 2. A total of 25 participants were initially screened, and 1 participant was excluded due to schedule conflicts. Twenty-four participants were successfully recruited. It included 17 healthy adult participants (11 females, mean age 28.8 years old) and 7 cLBP patients (5 females, mean age 44.4 years old). With an average of 14 years of pain duration, cLBP patients reported higher pain intensity and interference (Brief Pain Inventory; intensity, 5.0 ± 1.4; interference, 3.7 ± 2.5) than healthy participants (intensity, 0.3 ± 0.4; interference, 0.1 ± 0.2).

Table 3 includes the sample size and time lengths of various session durations: baseline, the time from session start to pressure threshold, the time from pressure threshold to tolerance, pinprick, and cuff. The time variance among pressure threshold and pressure tolerance sessions is high, with standard deviations (STD) of 3.74 and 5.34, respectively, compared to their mean values of 6.86 and 13.99, respectively. The time lengths of pinprick and cuff sessions were more stable regarding the variance, with STDs of 1.44 and 3.39, respectively, compared to their mean values of 6.99 and 29.95, respectively.

### 3.1. Analysis Plan 1—Optimal Time Window Analysis

**Figure 3:**
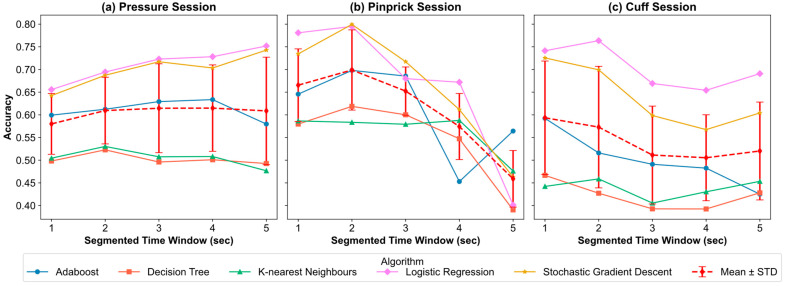
Accuracy curve of all algorithms and the mean accuracy of five algorithms under five segmented time windows (1, 2, 3, 4, 5 s) among pressure session (**a**), pinprick session (**b**), and cuff session (**c**). The red line in each figure shows the mean and standard deviation (STD) of all algorithms under different segmented time windows.

Figure 3 shows three accuracy curve plots of three sessions based on different algorithms and five segmented time windows. For each session, the time window selected was about three factors: accuracy of classification, time variance, and size of the datapoints.

For the pressure session, the highest average performance was achieved when the time window was set as 3 s (accuracy = 61.4%, f-1 = 54.3%), followed by 4 s (accuracy = 61.4%, f-1 = 53.5%). However, the highest performance was achieved in the logistic regression classifier of the 5 s time window (accuracy = 75.2%, f-1 = 67.2%). The size of the datapoint generated from a 5 s time window is 558, smaller than that generated from a 3 s time window, which is 1112 datapoints. Considering all factors, 3 s was selected as the optimal time window for the pressure session.

For the pinprick session, the highest performance was achieved in the SGD classifier using a 2 s time window (accuracy = 79.9%, f-1 = 64.8%). The highest average accuracy was achieved with the 2 s time window (69.9%), followed by 1 s (66.5%), and 3 s (65.2%). The optimal time window for the pinprick session was 2 s.

For the cuff session, the highest performance was achieved when the time window was set as 2 s in the LOG classifier (accuracy = 76.4%), followed by the LOG classifier (accuracy = 74.1%) and SGD classifier (accuracy = 72.6%) under a 1 s time window. The highest average performance was achieved in the 1 s time window (accuracy = 59.3%, f-1 = 41.2%), followed by the 2 s (accuracy = 57.3%, f-1 = 41.2%). Considering the dataset size, 4478 datapoints were segmented using the 1 s time window before SMOTE, and 2047 datapoints were generated with the 2 s time window. Therefore, a 1 s time window was chosen for the cuff session.

### 3.2. Analysis Plan 2—Component Sensitivity Analysis

This plan investigated the optimal sensor set for all QST sessions. First, a baseline model that included features from all sensors was established. This model employed PCA and performed the classification model based on the best model from the previous section. The LOG model was identified as the optimal for machine learning for all three sessions because this model achieved the highest classification accuracy performance. Second, six additional sensor set plans were compared by iteratively removing one sensor at a time from the following set: pupillometry, BVP, EMG, GSR, RR, and ST.

**Figure 4:**
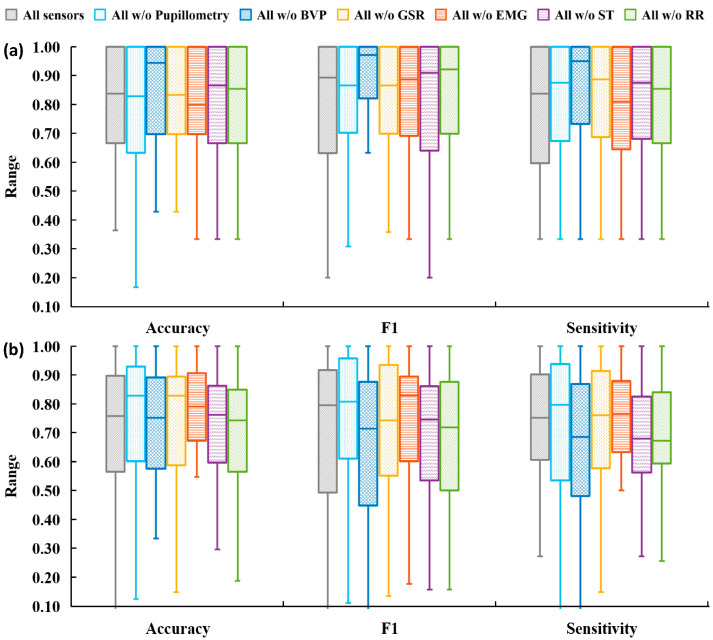
Performance metrics of the pinprick session and cuff session are presented in grouped boxplots (**a**,**b**). Each bar shows the mean and standard deviation.

For the pinprick session, the baseline model demonstrated an accuracy of 79.8% (f-1 = 62.9%). Six additional sets were evaluated, as shown in Table 4. The differences in accuracy compared to the baseline model are depicted in Figure 4a. The removal of BVP was found to significantly enhance overall performance, with increases of 6.2% in accuracy and 10.9% in f-1 (*p* < 0.05, Wilcoxon signed-rank test). Compared to the baseline, EMG and ST sensors had a negligible impact on the accuracy, with absolute accuracy differences of less than 1%. Removing GSR and RR sensors resulted in non-significant (*p* > 0.05, Wilcoxon signed-rank test) accuracy improvements of 2.3% and 2.2%, respectively. Excluding pupillometry decreased non-significant accuracy by 1.4%.

For the cuff session, the baseline model achieved accuracies of 76.5% (f-1 = 60.8%). The performance from the LOO analysis is shown in Table 4 and illustrated in Figure 4b. For the LOG classifier, both the BVP and RR were observed to significantly decrease in accuracy by 6.1% (*p* < 0.05) and 2.3% (*p* < 0.05), respectively. Removing EMG and ST led to 1.9% and 5.5% decreases in accuracy, respectively, while these changes were not statistically significant. Excluding GSR and pupillometry showed an increase in performance, with accuracy improvements of 2.5% (*p* < 0.05) and 5.3% (*p* < 0.05), respectively.

In contrast, the pressure session showed minimal variation in performance across different sensor sets in Table 4. The performance of the LOG model was stable and not affected by the removal of individual sensors. The removal of BVP, EMG, GSR, ST, and pupillometry decreased the performance, with less than 2% for the accuracy and f-1 score.

In summary, it was found that removing BVP significantly improved accuracy for the pinprick session, whereas removing EMG, GSR, RR, ST, and pupillometry did not significantly impact the classification performance. Regarding the cuff session, the elimination of BVP, GSR, RR, and pupillometry significantly impacted accuracy, while EMG and ST showed non-significant performance, indicating they were non-critical sensor candidates. None of the singular sensors significantly impacted the performance of the pressure sessions.

## 4. Discussion

This study represents a novel approach to objectively assess and classify pain intensity levels utilizing physiological sensors across pressure, pinprick, and cuff sessions. Our methodology involved classifying traditional subjective ratings, such as baseline, threshold, and tolerance in the pressure session, and categorizing pain intensity levels into mild, moderate, and severe pain in the pinprick and cuff sessions based on physiological features and multiple classification models to achieve optimal performance. Two critical analyses were explored: (1) determining the optimal segmented time window (ranging from 1 to 5 s); (2) identifying the individual contributions of each singular sensor by implementing an LOO iteration strategy and classifying critical and non-critical sensor candidates.

Existing literature on pain intensity level classification using physiological signals typically employed fixed time windows, such as 1 s [41], 4 s [42], and 10 s [23]. Our study contributes to this field by investigating five different time windows under three QST sessions. The results highlighted uniformity among baseline, pinprick, and cuff sessions but a significant variance across the pressure sessions. Such variability presented a challenge in determining the segmented time window. Our study carefully weighed the trade-offs among the factors like accuracy, F1 score, number of datapoints, and distributions of time lengths. For instance, a 2 s time window was chosen for the pinprick session due to its superior performance in classification models. In contrast, the cuff session’s time window was selected based on average performance and dataset size. However, the pressure session did not show significant performance differences between the 3, 4, and 5 s time windows. This highlights the need for discussing confounding factors such as psychosocial factors [13,43].

In analyzing optimal sensor sets for QST sessions, our baseline all-sensor models were compared against six other sets, each excluding one sensor iteratively. Our results reveal that removing BVP improved accuracy in pinprick sessions but decreased accuracy in cuff sessions. Removing EMG and ST sensors had negligible impact on pinprick session outcomes. In the cuff session, removing BVP and RR had negative impacts, whereas eliminating GSR and pupillometry significantly improved performance. Other literature analyzed the relationship between cuff sessions with singular physiological signals, such as EMG [35] and BVP [44], but very few published studies have examined the cuff pain intensity level classification via physiological signals. The pressure session presented uniformly consistent results, with an average accuracy variation of less than 2% among seven sensor sets using the LOG models. The reasons for this uniformity are not fully understood and warrant further investigation. Potential reasons include confounding factors such as the selection of time windows, the number of data points, and the participant population. The performance is consistent with one other study, which found that the highest performance of a three-class pain level classification in pressure pain session was achieved with 69% accuracy, 83.3% sensitivity, and 75% specificity [33].

Our study contributed to the field by indicating sensors (i.e., EMG and ST) that contributed minimally to classification performance and, as a result, implying a solution for cases when reducing sensor redundancy if necessary. Our study also highlighted the sensor that significantly impacted performance in both pinprick and cuff sessions (i.e., BVP), and sensors that are critical but stimuli-sensitive (i.e., GSR and RR). These stimuli-sensitive sensors should be further analyzed in sensor configuration tests. Pressure sessions demonstrated uniform performance, indicating that the pressure pain classification may rely on generalized physiological responses rather than specific sensor inputs.

The limitations of our study are multifaceted. First, the study’s approach of consolidating a limited and unbalanced sample of healthy participants (N = 17) and chronic patients (N = 7) into a single group was necessary for generalizability. At the same time, it limits the sensitivity of our findings between these distinct groups. Second, the limited specificity performance of non-critical sensors (i.e., EMG and ST in cuff sessions; EMG, GSR, RR, ST in pinprick sessions) does not directly mean that they can be excluded in all cases. Psychological, environmental, and physical activity factors might lead to limited-specificity performance. Alternative solutions can be achieved in multiple ways. One solution can be to integrate multimodal deep learning models like long short-term memory models and transfer learning [45,46]. Exploring different sensor fusion methods such as feature-fusion, decision-level fusion can also be an alternative way [47]. A new field, network physiology, can be integrated into the pain assessment problem [48]. Instead of evaluating sensors in a deterministic role, this area can treat sensors as probabilistic models to analyze the connectivity between each modality [49,50]. This holistic approach acknowledges the dynamic connections between different physiological modalities, potentially resolving inconsistencies where a sensor may be effective in one context but not in another [49,51].

Exploring individual variations in response to different stimuli is a promising area to understand pain sensitivity. The current practice of pain sensitivity is assessed by patient’s self-report, which cannot exclude the presence of inter- and intra-subject variability in characteristics such as psychological factors [13]. Stimulus-specific physiological responses represent a novel and critical area of exploration, linking specific physiological modalities to distinct noxious stimuli. This consideration is particularly important; when considering enhancing the portability and practicality of sensor configurations for chronic pain patients, sensor selection should be tailored to the specific type of pain being assessed. Future research can replicate this study following the described QST procedures, analysis plans, and pseudocode algorithms in the Supplementary Materials. This framework can be easily extended to explore the relationship between physiological responses to other dimensions of pain assessment, such as types of stimuli and pain locations, beyond pain intensity. The ability to identify the types and locations of pain will benefit patients who have difficulties in self-reporting [52].

In terms of broader impacts, this sensor sensitivity study paves the way for enhancing the portability and feasibility of pain assessment, especially in at-home settings. This study suggests that sensors having minimal impact on performance can be excluded from wearable pain assessment devices. This result can be used to simplify pain assessment device design by only including sensors such as GSR, BVP, and RR. That is to say, there are plenty of digital health technologies for remote data acquisition [53]. For example, the Empatica watch monitors BVP, GSR, and ST (Empatica, Empatica Inc., Cambridge, MA, USA); the Google Fitbit series collects different sets of sensors among PPG, oxygen saturation, GSR, and ST (Google, Santa Clara, CA, USA). By selecting sensor modalities sensitive to specific noxious stimuli, researchers can balance feasibility with model performance and enhance the practicality of remote pain assessment [26].

## 5. Conclusions

This study presented a novel framework for pain assessment using physiological sensors during QST sessions, integrating two complementary analysis plans. Analysis Plan 1 identified optimal time windows for signal segmentation, with 1–5 s windows yielding varied results across pinprick, cuff, and pressure sessions. The findings highlight the importance of tailoring time segmentation to specific stimuli to maximize classification performance. Analysis Plan 2 evaluated sensor contributions using leave-one-out iterations. BVP, GSR, RR, and pupillometry were identified as stimulus-specific critical sensor candidates, although only BVP showed significant performance across stimuli. In contrast, EMG and ST were found to be non-critical, showing minimal impact on performance across all sessions. Future research should explore stimulus-specific physiological responses further to optimize sensor configurations for different pain types. Incorporating advanced multi-sensor fusion techniques and individualization methodology can support the development of personalized, efficient, and practical wearable systems for chronic pain assessment and management.

## Figures and Tables

**Table 1:** Grid search hyperparameters of classifiers.

Logistic Regression	C	10^−3^, 10^−2^, 10^−1^, 1, 10, 10^2^, 10^3^
	Penalty	L1, L2
Decision Tree	Criterion	gini, entropy
	Max Depth	4, 5, 6, 7, 8, 9, 10, 11, 12, 15, 20, 30, 40, 50, 70, 90, 120
K Nearest Neighbors	Algorithm	Ball tree, kd tree, brute
	Leaf size	Range from 1 to 50 step 3
	N neighbors	10, 13, 16, 19, 22, 25, 28
Stochastic Gradient Descent	Alpha	10^−2^, 10^−3^, 10^−4^
	L1 ratio	0.05, 0.06, 0.07, 0.08, 0.09, 0.1, 0.12, 0.13, 0.14, 0.15, 0.2
	Penalty	L1, L2
	Loss function	hinge, log, modified Huber, squared hinge
AdaBoost	Base estimator	Decision tree
	Max depth	2, 5, 8, 11
	Min sample	5, 10
	N estimators	10, 50, 100, 250
	Learning rate	0.01, 0.1

**Table 2:** Demographic information.

Mean ± SD or %	cLBP Patient	Healthy Group
Number of participants	7	17
Age, y	44.4 ± 14.5	28.8 ± 13.1
Female sex	5	11
Pain duration, y	14.0 ± 15.5	0
Pain intensity	5.0 ± 1.4	0.3 ± 0.4
Pain interference	3.7 ± 2.5	0.1 ± 0.2

**Table 3:** Time length statistics of QST sessions.

QST Session	Sample Size	Mean ± STD (s)
Baseline	32	59.80 ± 6.93
Pressure–Threshold	160	6.86 ± 3.74
Pressure–Tolerance	160	13.99 ± 5.34
Pinprick	128	6.99 ± 1.44
Cuff	128	29.95 ± 3.39

**Table 4:** Performance of pinprick, cuff, and pressure sessions.

	Pinprick		Cuff		Pressure	
Sensor Set	Accuracy %	F-1 Score %	Accuracy %	F-1 Score %	Accuracy %	F-1 Score %
All sensors	79.8	62.9	76.5	60.8	72.3	66.4
All w/o BVP	86 ↑	73.8 ↑	70.4 ↓	47.5 ↓	72.3	66.4
All w/o EMG	80.7	65.4	74.6	58.6	72.3	66.4
All w/o GSR	82.1	67.7	79.0 ↑	60.7 ↑	72.3	66.4
All w/o RR	80.6	63.8	74.2 ↓	53.9 ↓	72.4	66.7
All w/o ST	80.7	67.4	71.0	51.1	72.3	66.4
All w/o pupillometry	78.4	59.9	81.8 ↑	62.3 ↑	72.3	66.4

↑↓ indicates a statistically significant increase or decrease in performance (Wilcoxon signed-rank, *p* < 0.05).

## Data Availability

Datasets in the study are available from the corresponding author upon reasonable request.

## Supplementary Materials

The following supporting information can be downloaded at: https://www.mdpi.com/article/10.3390/s25072086/s1, File S1: Supplementary Material: Pseudocode Algorithms for Physiological Sensor Modality Sensitivity Test for Pain Intensity Classification in Quantitative Sensory Testing.
