# Supplementary material for: Physiological Sensor Modality Sensitivity Test for Pain Intensity Classification in Quantitative Sensory Testing
Source: Sensors (Basel). 2025 Mar 26;25(7):2086. doi: 10.3390/s25072086 (PMC11991361; doi:10.3390/s25072086)
Supplement: Supplementary file 1 [file sensors-25-02086-s001.zip › sensors-3454968-supplementary.pdf]

Supplementary Materials

Pseudocode Algorithms for Physiological Sensor Modality Sensitivity Test for Pain Intensity Classification in Quantitative Sensory Testing

This supplementary material presents two pseudocode algorithms used in Analysis Plan 1 and Analysis Plan 2, respectively. The first algorithm, *Time Window Optimization and Classification*, is to determine optimal time window and classifier parameters for pain intensity classification. The second algorithm, *Sensor Sensitivity Analysis via Leave-One-Out*, aims at evaluating individual sensor contributions to classification performance. Both algorithms are programmed in Python environments.

Algorithm S1: Time Window Optimization and Classification Pipeline

```
FUNCTION func_pca_dt(df, dataset_path):
    // Data preprocessing
    imputer = SimpleImputer(strategy='mean')
    df_filled = impute missing values in df using imputer
    replace infinite values with NaN in df_filled
    remove rows with NaN from df_filled

    // Feature scaling
    scaler = StandardScaler()
    scaled_features = scaler.fit_transform(df_filled)

    IF scaled_features contain NaN or infinite values:
        RAISE error

    // Dimensionality reduction
    pca = PCA(n_components=preset threshold)
    principal_components = pca.fit_transform(scaled_features)
    pca_df = create DataFrame with principal_components
    pca_df add 'class_label' from original df

    // Get important features
    explained_variance = pca.explained_variance_ratio_
    cumulative_variance = cumulative sum of explained_variance
    num_components = pca.n_components_

    FOR each principal component:
        find most important feature
```

```
    store component feature and variance

// Model training/evaluation
subjects = unique subjects from df
classifier_configs = [
    (DecisionTree, {parameter grid}),
    (KNN, {parameter grid}),
    (SGD, {parameter grid}),
    (LogisticRegression, {parameter grid}),
    (AdaBoost, {parameter grid})
]

FOR each (classifier, params) in classifier_configs:
    accuracies = empty list

    FOR each subject in subjects:
        // Data splitting
        test_data = pca_df entries for current subject
        train_data = pca_df entries excluding current subject

        X_train, y_train = train data without class labels
        X_test, y_test = test data without class labels

        // Handle class imbalance
        TRY:
            apply SMOTE to X_train, y_train
        EXCEPT:
            skip SMOTE

        // Hyperparameter tuning
        grid_search = RandomizedSearchCV(classifier, params)
        grid_search.fit(X_train, y_train)
        best_params = grid_search best parameters
        best_estimator = grid_search best model

        // Evaluation
        y_pred = best_estimator.predict(X_test)
        accuracy = calculate accuracy(y_test, y_pred)
        append accuracy to accuracies
    mean_accuracy = average of accuracies
```

```
        print mean_accuracy

// Main execution
FUNCTION PerformTimeWindowAnalysis():
    // Time window configuration
    FOR EACH time_window IN list_timewindow:
        CREATE segmentation folder path using time_window
        SET durations for BL/PP/Cuff phases equal to time_window

        // Data segmentation
        dict_segments = EMPTY DICTIONARY

        // Process physiological signals
        FOR EACH subject IN physiological_data:
            FOR EACH experimental_phase IN [BL, Thresh, Tolerance, PPks, Cuff]:
                DETERMINE phase duration based on experimental_phase type
                SET initial segment end = phase_end_timestamp

                // Create overlapping segments
                WHILE segment start > phase_start_timestamp:
                    EXTRACT data vectors for:
                    - BVP
                    - EMG
                    - Skin Conductance
                    - Temperature
                    - Respiration
                    - EDA components
                    STORE in dict_segments with composite key:
                    [subject]-[phase]_[segment#]_pain_[level]

        // Process eye tracking data
        FOR EACH subject IN eye_tracking_data:
            FOR EACH experimental_phase IN [BL, Thresh, Tolerance]:
                DETERMINE phase duration
                EXTRACT eye movement features:
                INTEGRATE with physiological segments

        // Feature extraction
        df_features = GENERATE_FEATURES(dict_segments):
            FOR EACH segment IN dict_segments:
```

```
        CALCULATE temporal features:
        - Mean/Std of physiological signals
        - Frequency domain features
        - EDA decomposition features
        - Eye movement features
        APPEND pain_level label from experimental design

    // Execute classification pipeline
    CALL func_pca_dt(df_features)
```

Algorithm S2: Sensor Sensitivity Analysis via Leave-One-Out

```
FUNCTION PerformSensorSensitivityAnalysis():
  // Sensor feature groups
  SENSOR_FEATURES = {
    'none': [],  // Control group (all features)
    'bvp': PPG Features,
    'sc': EDA Features,
    'temp': Thermal Features,
    'resp': Respiratory Features,
    'eye': Eye movement Features,
    'emg': emg Features
  }
  // Load and prepare dataset
  pain_data = LOAD_CSV(pressure/pinprick/cuff_path)
  FILTER baseline records
  CREATE class_labels using experimental protocol

  // Sensor exclusion loop
  FOR EACH (sensor_group, excluded_features) IN SENSOR_FEATURES:

    // Create feature subset
      reduced_data = pain_data.DROP_COLUMNS(excluded_features)
    // Validate reduced feature set
    RUN_CLASSIFICATION_PIPELINE(
      data = reduced_data,
      output_path = dataset_path,
      config_name = sensor_group
    )
```
